# Increased Mortality Rates During the 2025 Chikungunya Epidemic in Réunion Island

**DOI:** 10.3390/v18020180

**Published:** 2026-01-29

**Authors:** André Ricardo Ribas Freitas, Luana Hughes Freitas, Antonio Silva Lima Neto, Luciano Pamplona Goes Cavalcanti, Pedro María Alarcón-Elbal

**Affiliations:** 1 Curso de Medicina, Faculdade São Leopoldo Mandic, Instituto de Pesquisa São Leopoldo Mandic, Campinas 13045-755, SP, Brazil; 2 Faculdade de Engenharia de Computação, Insper, Vila Olímpia, São Paulo 04546-042, SP, Brazil; luanahf1234@gmail.com; 3 Centro de Ciências da Saúde, Universidade de Fortaleza, Fortaleza 60811-000, CE, Brazil; tanta26@yahoo.com; 4 Secretaria de Saúde do Ceará, Fortaleza 60000-000, CE, Brazil; 5 Faculdade de Medicina, Centro Universitário Christus, Fortaleza 60160-000, CE, Brazil; pamplona.luciano@gmail.com; 6 Faculdade de Medicina, Universidade Federal do Ceará, Fortaleza 60000-000, CE, Brazil; 7 Department of Animal Production and Health, Public Veterinary Health and Food Science and Technology, Faculty of Veterinary Medicine, Universidad Cardenal Herrera-CEU, CEU Universities, 45115 Valencia, Spain; pedro.alarconelbal@uchceu.es

**Keywords:** chikungunya virus, excess mortality, underreporting, arboviruses, public health surveillance, Réunion Island, emerging infectious diseases

## Abstract

Background: Chikungunya virus (CHIKV) has historically been regarded as a low-fatality infection; however, growing evidence from diverse study designs demonstrated a substantial mortality burden during large-scale epidemics. In 2025, Réunion Island experienced a major CHIKV outbreak, raising renewed concerns about its fatal impact. Methods: We conducted an ecological time-series analysis of all-cause mortality during the 2025 chikungunya epidemic. Expected deaths were estimated using two complementary approaches: (i) a baseline based on age-specific mean mortality rates from the same calendar months in the post-pandemic period and (ii) long-term Poisson regression models using a log-link function and population offset, excluding the COVID-19 pandemic period. Excess mortality was calculated as the difference between observed and expected deaths during periods when observed mortality significantly exceeded the upper bound of the 95% confidence interval (CI). Results: Observed mortality exceeded the upper 95% CI limit for three consecutive months, coinciding with the epidemic curve and resulting in an estimated 208 excess deaths. These deaths were concentrated among older adults, peaking in April 2025 with a mortality rate ratio of 1.34 (95% CI: 1.22–1.47; *p* < 0.001). Among older adults, the age-specific excess mortality rate reached 145.3 per 100,000 (95% CI: 125.5–165.0) with a case fatality rate (CFR) of 2.4%, resulting in an overall population excess mortality rate of 23.2 per 100,000 and a total CFR of 0.4%. The number of deaths identified through routine surveillance was substantially lower than our estimates, highlighting a significant discrepancy between reported and excess chikungunya-associated mortality. Conclusions: Chikungunya epidemics are consistently associated with substantial underrecognized mortality worldwide. Routine surveillance relying solely on laboratory confirmation underestimates the true burden of the disease. Integrating excess mortality analysis, strengthening diagnostic and postmortem investigations, and implementing timely mitigation measures are essential to accurately assess and reduce preventable deaths during future CHIKV outbreaks.

## 1. Introduction

Chikungunya virus (CHIKV) remains a major global public health challenge, particularly in regions where competent vectors and susceptible populations coexist. Transmitted primarily by *Aedes aegypti* and secondarily by *Aedes albopictus*, the virus has demonstrated a remarkable ability to adapt to different ecological settings [1,2]. Although most infections are self-limited, increasing evidence from diverse study designs indicates that CHIKV infection is not uniformly benign and may be associated with severe outcomes and death, particularly among older adults and individuals with underlying conditions. Plausible pathways include severe acute disease with systemic inflammation and multi-organ dysfunction, myocarditis and malignant arrhythmias, neurological complications such as encephalitis, acute kidney injury, and indirect deaths due to decompensation of pre-existing cardiometabolic and chronic diseases during or shortly after acute infection [3,4,5], challenging the long-standing notion that chikungunya is a benign infection [5,6,7]. In the first decade of this century, large outbreaks occurred in areas with the exclusive presence of *A. albopictus*, most notably in Réunion (a tropical French territory in the Indian Ocean), while autochthonous transmission was also documented in mainland Europe, though without large-scale outbreaks. These events were facilitated by a viral lineage carrying the A226V mutation in the E1 glycoprotein, which enhanced transmission efficiency by this vector [1,2]. This same mutation, along with other genetic changes, has been identified in the lineage currently circulating in Réunion Island (2024–2025) [8]. Climatic factors—particularly the exceptionally hot summer this year [9]—combined with the widespread presence of *Aedes albopictus* and the circulation of this adapted viral lineage may be contributing to the unusually early and simultaneous transmission observed in multiple parts of Europe and in China in 2025 [10,11].

The resurgence of CHIKV transmission in Réunion Island in mid-2024 and into 2025 has renewed concerns about the disease’s mortality burden. By the end of June 2025, a total of 54,250 biologically confirmed cases had been reported. During the same period, 27 deaths were attributed to chikungunya by the official causality assessment committee—17 classified as directly related and 10 as indirectly related—while 26 additional deaths remained under investigation [12]. The study aims to quantify all-cause excess mortality during the 2025 chikungunya epidemic in Réunion, based on official mortality data from the INSEE (Institut National de la Statistique et des Études Économiques), using different statistical approaches.

## 2. Materials and Methods

To estimate expected deaths during the 2024–2025 chikungunya epidemic in Réunion Island, we used complementary baseline strategies that differ in their reliance on modeling assumptions and in the time window used to define “non-epidemic” mortality. First, we applied a parsimonious recent-years approach based on the two most recent pre-epidemic years (Section 2.2), designed to provide a transparent, assumption-light estimate reflecting contemporary mortality patterns. Second, we fitted long-term Poisson regression baselines to the 2010–2025 time series (excluding 2020–2022 due to the COVID-19 impact) to explicitly account for secular trends and seasonality (Section 2.3). Within this long-term framework, we implemented two alternative seasonal specifications—a Serfling-type harmonic model and a model treating month as a nominal categorical variable—to evaluate robustness to seasonal-model choice. Concordant findings across approaches were interpreted as evidence that results were not driven by baseline specification.

### 2.1. Source and Preparation of Mortality and Population Data

#### 2.1.1. Data Sources

All data were obtained from publicly available official sources. We used secondary mortality data obtained from INSEE [13], the official source for national mortality statistics in France. With comprehensive and up-to-date mortality data, the INSEE system is an effective tool for tracking fluctuations in overall mortality in both epidemics and extreme heat waves [9]. Chikungunya case and death notifications were sourced from Santé Publique France [https://www.santepubliquefrance.fr, accessed on 2 July 2025] [12].

#### 2.1.2. Preparation of Mortality and Population Data

Mortality data covered the period from January 2010 to May 2025 and included all-cause mortality in La Réunion, disaggregated by month and age group. For the purposes of this analysis, mortality data were stratified into three age groups: <25 years, 25–64 years, and ≥65 years. Monthly population estimates were derived by geometric interpolation based on annual population figures provided by INSEE [14].

### 2.2. Age-Specific Expected Deaths Based on Post-Pandemic Baseline

Considering that the COVID-19 pandemic ended in August 2022, monthly baseline mortality rates were defined using observed mortality data from September 2022 to May 2024. This recent-years baseline was used as a transparent, assumption-light reference and was complemented by long-term Poisson regression baselines in Section 2.3. For each month *m* and age group, the baseline mortality rate (*λ_m_*) was calculated as

$$\lambda_{m}=\frac{D_{m,y_{1}}+D_{m,y_{2}}}{P_{m,y_{1}}+P_{m,y_{2}}}$$

where *D_m_*_,__*γ*_ represents the number of deaths and *P_m_*_,__*γ*_ the population for month *m* in year *y*.

Data from 2022–2023 were used for September–December 2024, and from 2023–2024 for January–May 2025. February 2024 counts were standardized to 28 days to account for the leap year. Expected deaths (*E_m_*) for months in 2024–2025 were then estimated as

$$E_{m}=\lambda_{m}\times P_{m,2024-25}$$

The incidence rate ratio (*IRR_m_* defined as the ratio of observed to expected monthly death) was computed as

$$IRR_{m}=\frac{O_{m}}{E_{m}}$$

where *O_m_* denotes the observed deaths in month *m*.

Ninety-five percent confidence intervals (CIs) for *IRR_m_* were derived under a Poisson distribution assumption using both exact methods and Byar’s approximation. Statistical significance was assessed under the null hypothesis that

$$O_{m}∼\mathrm{Poisson}\left(E_{m}\right)$$

Two-tailed *p*-values were calculated to detect deviations in mortality (increase or decrease), and one-tailed *p*-values to specifically detect increases in mortality. Months were considered to exhibit significant excess mortality only when the lower bound of the 95% CI for *IRR_m_* exceeded 1.00 and the one-tailed *p*-value was below 0.05. Excess mortality was computed as

$$\mathrm{Excess}\;{\mathrm{Mortality}}_{m}=O_{m}-E_{m}$$

Analyses were performed in Python (version 3.13, 64-bit) using the libraries *numpy*, *pandas*, *scipy.stats*, and *statsmodels*.

### 2.3. Long-Term Mortality Modeling Using Poisson Regression (2010–2025)

To complement the recent-years baseline approach described in Section 2.2 and construct a robust baseline for expected mortality during the chikungunya epidemic, we employed Poisson regression models that account for long-term trends (2010–2025), seasonal variability, and population growth. The years 2020 to 2022 were excluded from the baseline estimation due to the impact of the COVID-19 pandemic on mortality patterns [14]. This baseline intentionally incorporates background seasonal mortality fluctuations potentially associated with influenza, other infectious, and environmental drivers of seasonal fluctuations, ensuring that any unusual mortality patterns during the chikungunya epidemic are detected against a conservative, real-world reference level.

Two modeling strategies were used: (1) a Serfling-type seasonal regression model incorporating Fourier harmonics to capture periodic fluctuations in mortality, and (2) an alternative approach using month as a nominal categorical variable to flexibly model intra-annual variation without assuming a specific seasonal pattern. In both models, a log-transformed population offset was included to adjust for differences in population size over time.

#### 2.3.1. Serfling-Type Regression, Harmonic Seasonality

To construct the Serfling model, we standardized monthly death counts to a 30-day month to ensure comparability across months and over time. We used monthly terms instead of weekly ones, as commonly done in systems like EuroMOMO, because the INSEE database did not provide the exact date of death (only month and year) for the period 2010 to 2017 [13,15,16]. We applied a generalized linear model with a Poisson distribution and a log-link function. The model incorporated a linear time trend (month number) and seasonal components via harmonic terms to capture both annual and sub-annual periodicities, which are particularly important in tropical and subtropical regions [15,17,18,19,20,21]. Following the principle of parsimony, we sequentially tested models including one to four seasonal harmonics Fourier terms. The final model included the linear trend and all seasonal harmonics, which provided an adequate fit to the observed seasonal variation. For the ≥65 years age group, the full model was not only the best-fitting specification according to multiple criteria (deviance, AIC, and BIC), but all harmonic terms were statistically significant, highlighting the importance of capturing detailed seasonal variation in this subgroup. To ensure consistency across age groups, we adopted the same model structure for all strata, including the linear time trend and sine and cosine terms up to the fourth harmonic to ensure comparability of trends across groups along. The final model equation was specified as

$$\log\left(E\left(Y_{t}\right)\right)=\beta_{0}+\beta_{1}t+\sum_{k=1}^{4}\left[\beta_{2k}\sin\left(\frac{2\pi kt}{12}\right)+\beta_{2k+1}\cos\left(\frac{2\pi kt}{12}\right)\right]+\log\left(P_{t}\right)$$

**where**

*E(Y_t_): expected number of deaths at month t;*

*t: time in months;*

*β_0_: intercept;*

*β_1_: linear time trend coefficient;*

*β_2k_, β_2k+1_: seasonal harmonic coefficients;*

*P_t_: population at risk (offset).*

#### 2.3.2. Alternative Model Using Categorical Month Indicators

As an alternative seasonal specification within the long-term Poisson framework, we also tested an alternative and well-established approach using a Poisson regression model in which the month was treated as a categorical (nominal) variable [15,21,22]. This method captures seasonal effects by assigning a distinct baseline to each calendar month, without assuming a sinusoidal pattern. This allows for greater flexibility in detecting deviations from expected mortality trends, particularly when mortality fluctuations do not follow a strictly harmonic structure.

The final model equation, with month as a nominal variable, was specified as

$$\log\left(E\left(Y_{t}\right)\right)=\beta_{0}+\beta_{1}\cdot t+\sum_{m=1}^{12}\beta_{m}\cdot M_{m}$$

**where**

*E(Y_t_) is the expected number of deaths at time t,*

*β_0_ is the intercept,*

*β_1_ is the coefficient for the linear time trend,*

*β_m_ represents the coefficients for each month (M_m_) and is treated as a nominal variable, with m = 1, 2, …, 12, representing each month of the year.*

**Excess Mortality Calculation and Detection Criteria**

To estimate excess mortality, we applied the fitted Poisson regression models to the full time series, including the epidemic period (February–May 2025), generating expected mortality values under non-epidemic conditions. Excess mortality was then calculated by comparing observed deaths to these model-based predictions.

To stabilize the variance and approximate normality in mortality counts, we applied a Box–Cox power transformation with exponent 2/3, as recommended by Farrington et al. (1996) [23] and adopted by the EuroMOMO project [24]. This transformation was applied to both observed and expected deaths, and computed the residuals in the transformed scale:

$$R_{t}=O_{t}^{2/3}-E_{t}^{2/3}$$

where *O_t_* is the observed and *E_t_* the expected death count for month *t*.

To estimate the standard deviation (SD) of these residuals, we used only the residuals from non-epidemic months, thereby avoiding inflation of baseline variability. Z-scores were then calculated as

$$Z_{t}=\frac{O_{t}^{2/3}-E_{t}^{2/3}}{SD}$$

Consistent with the EuroMOMO methodology [24], a Z-score > 2 was considered indicative of statistically significant excess mortality. Periods with sustained Z-scores above this threshold were interpreted as excess mortality events potentially related to the chikungunya epidemic.

Both Poisson regression analyses were conducted using IBM SPSS Statistics, version 24 (IBM Corp., Chicago, IL, USA).

## 3. Results

The analysis revealed a clear temporal association between the chikungunya epidemic and increased mortality in older adults on Réunion Island in 2025. Overall, mortality patterns remained stable for individuals under 65 years, while a marked surge in deaths was observed among those aged ≥65 years between March and May 2025.

Table 1 presents the observed and expected monthly deaths for three age groups (<25, 25–64, and ≥65 years) from September 2024 to May 2025, along with corresponding CIs, excess deaths, IRR_m_, and *p*-values., No statistically significant excess mortality was observed in any month among individuals under 65 years old.

**Figure 1:**
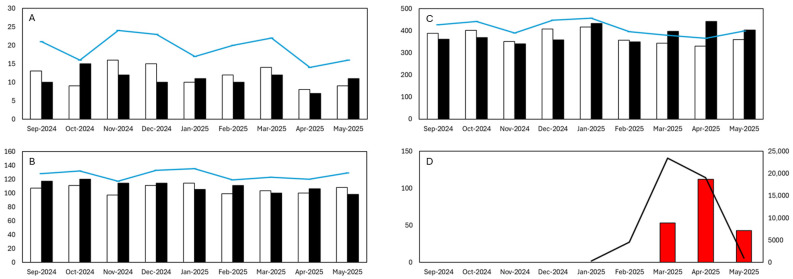
Monthly observed deaths (black bars), expected deaths based on the two most recent pre-epidemic years (Section 2.2; white bars), and the upper 95% confidence bound under the Poisson-based incidence IRRm framework (blue line), Réunion Island (France), September 2024–May 2025. Excess deaths are shown as red bars only for months with statistically significant excess mortality, defined as months in which observed deaths exceeded the upper 95% confidence limit of expected deaths. (**A**) Individuals aged 0–24 years. (**B**) Individuals aged 25–64 years. (**C**) Individuals aged ≥65 years. (**D**) Confirmed CHIKV cases (black line, right Y-axis) overlaid with excess deaths across all ages (red bars, left Y-axis). Deaths are plotted on the left Y-axis and confirmed CHIKV cases are plotted on the right Y-axis. An increase in mortality, especially among older adults, coincided with the peak of the chikungunya epidemic in March and April 2025.

In contrast, for individuals aged 65 years and older, a marked increase in mortality was detected during the chikungunya epidemic period. The overall estimated excess mortality for individuals aged ≥65 years during this three-month period was 208 deaths. The peak occurred in April 2025, with an IRR_m_ of 1.34 (95% CI: 1.22–1.47; *p* < 0.001), indicating a strong deviation from baseline mortality. Statistically significant excess mortality was also detected in March (53 excess deaths, IRR_m_ = 1.16, *p* < 0.01) and May (43 excess deaths, IRR_m_ = 1.12, *p* < 0.05), reinforcing the temporal alignment between the epidemic curve and increased deaths among older adults (Figure 1; Table 1).

Based on a population of 143,188 aged ≥65 years, the excess mortality rate was 145.3 per 100,000 (95% CI: 125.5–165.0. Since no excess deaths were observed among individuals aged 0–24 or 25–64 years, despite there being 18,090 and 27,506 reported chikungunya cases in these groups, respectively, the overall excess mortality rate for the total population (897,609) was 23.2 per 100,000. When adjusted for age using the French national population structure, the age-standardized excess mortality rate was 27.5 per 100,000 (95% CI: 23.7–31.2). Assuming homogeneous incidence across age groups, the estimated case fatality rate (CFR) among older adults reached 2.4%, compared to 0.4% overall.

**Figure 2:**
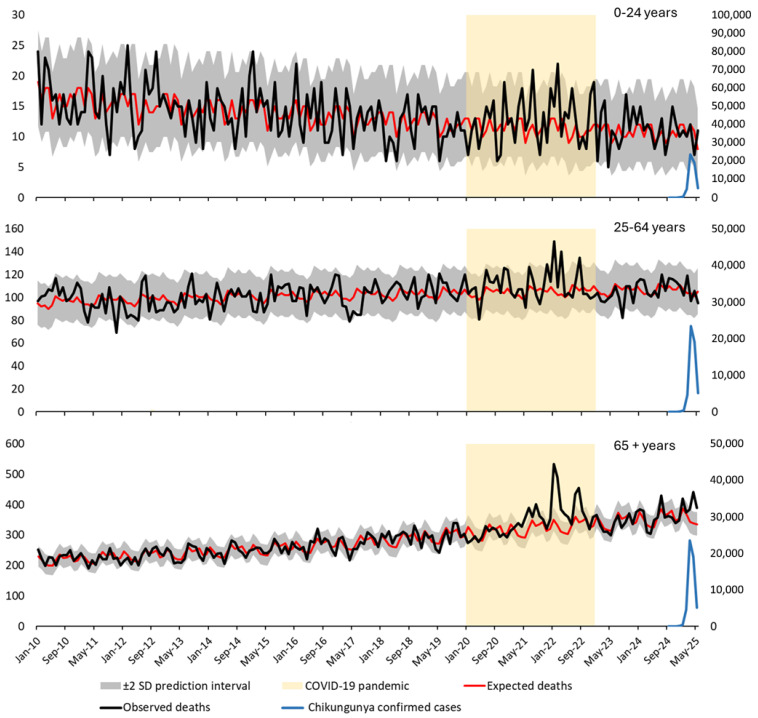
Time series of observed and expected all-cause mortality and confirmed chikungunya cases in Réunion Island (France), 2010–2025, stratified by age group, using Poisson regression with seasonal Fourier terms (Serfling-type model). This figure shows monthly time series of all-cause mortality and confirmed chikungunya cases in Réunion Island, stratified by age group: Top panel—individuals aged 0–24 years; Middle panel—individuals aged 25–64 years; Bottom panel—individuals aged ≥65 years. Observed mortality (black line) is plotted against expected mortality (red line). The shaded area represents the 95% prediction interval (±2 standard deviations). The yellow highlighted period corresponds to the COVID-19 pandemic (2020–2022). Confirmed monthly chikungunya cases (blue line, right Y-axis) are overlaid to illustrate the temporal relationship with mortality trends.

**Figure 3:**
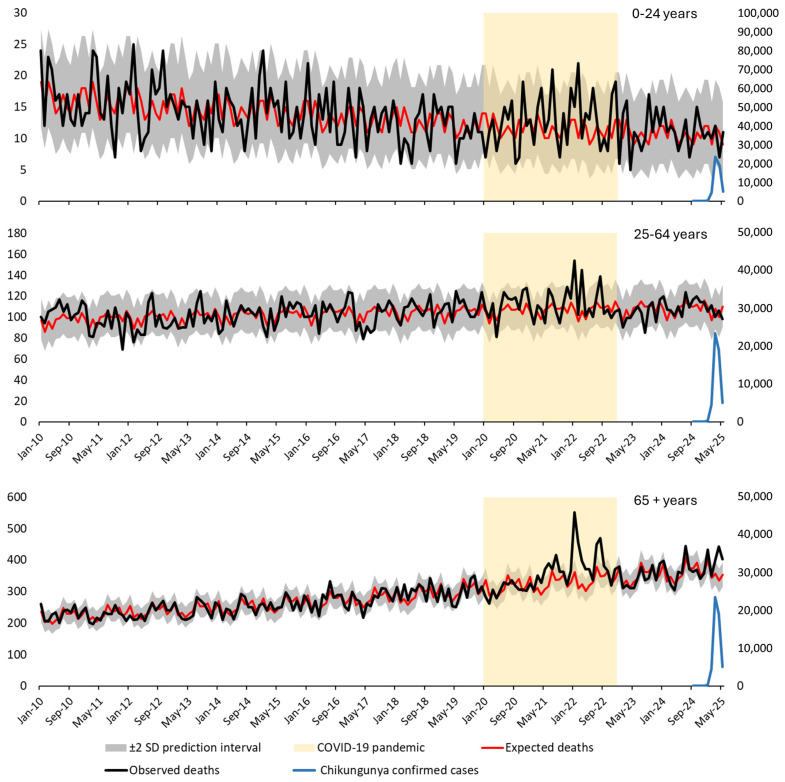
Time series of observed and expected all-cause mortality and confirmed chikungunya cases in Réunion Island (France), 2010–2025, stratified by age group using Poisson regression with categorical month terms. This figure shows monthly time series of all-cause mortality and confirmed chikungunya cases in Réunion Island, stratified by age group: Top panel—individuals aged 0–24 years; Middle panel—individuals aged 25–64 years; Bottom panel—individuals aged ≥65 years. Observed mortality (black line) is plotted against expected mortality (red line). The shaded area represents the 95% prediction interval (±2 standard deviations). The yellow highlighted period corresponds to the COVID-19 pandemic (2020–2022). Confirmed monthly chikungunya cases (blue line, right Y-axis) are overlaid to illustrate the temporal relationship with mortality trends.

Figure 2 shows the results of a Poisson regression model incorporating seasonal Fourier terms, following a Serfling-type approach. This method assumes a sinusoidal seasonal pattern to estimate baseline mortality, fitted to pre-epidemic data. Figure 3 presents the same data modeled using Poisson regression with categorical month terms as fixed effects. This alternative method captures seasonal variation without imposing a predefined functional form, allowing each calendar month to contribute independently to the expected mortality pattern.

In both figures, observed mortality (black line) is plotted against expected mortality (red line), with 95% prediction intervals (±2 standard deviations; shaded area). Confirmed monthly chikungunya cases (blue line, right Y-axis) and the COVID-19 pandemic period (yellow shaded area) are also shown for context. A marked mortality peak is observed in early 2025—particularly among individuals aged ≥65 years—consistently exceeding the upper prediction bounds in both models.

Table 2 presents monthly observed and expected deaths, 95% confidence intervals, and Z-scores for the three age groups in Réunion Island from September 2024 to May 2025 under both modeling strategies. Substantial and sustained excess mortality was observed only among individuals aged ≥65 years between March and May 2025, with Z-scores consistently exceeding 2. The estimated excess deaths in this group reached 202 under the Serfling-type model and 198 under the nominal-month model, confirming the robustness of the findings.

## 4. Discussion

Our findings demonstrated a clear temporal and spatial association between the chikungunya epidemic in Réunion Island in 2025. The overall estimated excess mortality for individuals aged ≥65 years during this three-month period was 208 deaths. We observed a marked increase in mortality among older adults, with minimal impact in younger age groups. The highest impact was observed in April 2025, with a mortality rate ratio of 1.34 (95% CI: 1.22–1.47; *p* < 0.001) in this age group. The observed excess deaths occurred in a narrow three-month window that coincided with the peak of the chikungunya epidemic, suggesting a direct or indirect link between viral transmission and increased mortality risk in vulnerable populations. The estimated case fatality rate (CFR) among older adults was 2.4%, compared to 0.4% in the general population. However, this figure likely underestimates the true CFR in the ≥65 age group. Given that many individuals in this cohort were likely exposed during the 2005–2006 epidemic and may have acquired long-lasting immunity, their risk of infection during the 2025 outbreak may have been lower than in younger groups. As the number of infections constitutes the denominator in CFR calculations, a reduced infection count in this group would result in an even higher actual CFR, further reinforcing the disproportionate vulnerability of older adults to severe and fatal outcomes of chikungunya.

This study employed various analytical methods, ranging from a simple approach based on mean post-pandemic age-specific mortality rates to dual Poisson regression strategies. These methodologies align with robust statistical modeling protocols from international agencies, including the World Health Organization (WHO), EuroMOMO, and the European Centre for Disease Prevention and Control (ECDC), and are commonly used for analyzing mortality associated with influenza, COVID-19, and natural disasters [9,15,16,21]. The consistency across these methods strengthens the robustness of our findings.

Excess mortality associated with chikungunya epidemics is not a new observation. One of the earliest signals comes from Calcutta during the 1963 outbreak of “acute hemorrhagic fever”, which occurred in a context where dengue was already endemic and expected to recur, whereas chikungunya was regarded as newly introduced (and possibly imported) into India [25]. In August–November 1963, deaths attributed to “unknown fever” increased markedly (218 observed vs. 60 expected), with the peak month (November) temporally aligning with the period in which chikungunya became the predominant virological finding [26]. Virological investigations isolated 36 viruses from acute sera, of which 35 were identified as chikungunya, while only one Group B isolate (i.e., a flavivirus, closely related to dengue-2) was obtained; importantly, the investigators concluded that most cases in September–October were associated with Group B infections, whereas cases in November and later were almost always associated with chikungunya, consistent with a two-wave pattern in which the chikungunya-associated wave built up by September but peaked in November [25,26,27]. Although dengue circulation cannot be excluded—given known challenges in dengue virus isolation and the possibility of mixed infections—the convergence of (i) excess deaths, (ii) the shift toward chikungunya predominance, and (iii) the November peak in chikungunya activity strengthens the plausibility that chikungunya contributed substantially to the observed mortality burden. Notably, Gelfand emphasized that “*During this period of time, no other epidemic of infectious disease was known to be occurring in Calcutta*” [26]. This early evidence already pointed to a higher-than-expected lethal potential for chikungunya outbreaks—a risk that has since been repeatedly underestimated. In multiple countries, large discrepancies between excess mortality estimates and officially reported deaths have been observed: in the Dominican Republic (2014), more than 800 times more deaths were estimated than reported; in Puerto Rico (2014), 1310 excess deaths contrasted with only 31 laboratory-confirmed fatalities; and in several Brazilian states (Bahia 2015–2016, Pernambuco 2016, Rio Grande do Norte 2016, Minas Gerais 2023), substantial excess mortality was documented despite very few confirmed deaths [22,28,29]. During a severe epidemic in India (2006), thousands of excess deaths and several fatal laboratory-confirmed cases were published in peer-reviewed journals, yet no deaths were officially reported [30,31]. Similar underreporting occurred in Jamaica (2014) and Mauritius (2006), where thousands of excess deaths were observed, but none were captured by official surveillance systems [32,33].

Excess mortality analysis has long been recognized as fundamental for accurately estimating the true impact of epidemic diseases. As early as the 19th century, Jacques Bertillon’s seminal investigation of the 1890 influenza pandemic in Paris demonstrated that deaths officially attributed to influenza vastly underestimated the outbreak’s toll [34]. By comparing observed mortality during epidemic peaks to a pre-pandemic baseline, he estimated at least 5000 influenza-attributable deaths—yet only 243 of these were certified as such by examining physicians. Similar discrepancies were documented in other European capitals, including London and Berlin [34]. Since the 1970s, the WHO has recommended using excess-death estimates to capture the true burden of influenza and to guide decisions on appropriately scaled control measures; these methods have since been extended to RSV, the COVID-19 pandemic, environmental disasters, and other high-impact events [21,35,36]. Over the last two decades, Santé Publique France has systematically monitored heatwaves and rapidly estimated excess deaths to support risk communication and guide preventive actions [37]. During the June–July 2025 heatwave, authorities reported 480 excess deaths—predominantly among older adults—underscoring their expertise and transparency in mortality surveillance [18]. The overall excess mortality rate for the total population was 23.2 per 100,000. These values are notably consistent with estimates from similar studies using excess mortality approaches, such as those reported by Josseran during the 2006 Réunion epidemic (33.8 per 100,000) and our previous work in Brazil during the 2023 epidemic (32.3 per 100,000) [22,38,39]. Additional comparisons with other chikungunya outbreaks are presented in the Supplementary Materials, Despite differences in geography, demographics, and healthcare infrastructure, these figures reveal a recurring mortality pattern associated with intense chikungunya transmission. This consistency across distinct settings supports the robustness of excess mortality analysis as a tool for quantifying the hidden impact of chikungunya and underscores the need for its systematic integration into routine epidemic surveillance and risk assessment frameworks [40].

The experience of French Overseas Territories—including Réunion (2005–2006) and the French Caribbean in 2014—and Brazil, among other countries, during chikungunya epidemics demonstrated that, despite substantial investments in healthcare and research, resulting in numerous peer-reviewed publications, officially confirmed deaths accounted for only a fraction of total excess mortality [28,39,41,42]. In addition to ecological excess-mortality analyses, large population-based studies utilizing individualized data also support an increased risk of death following chikungunya. In Brazil, linkage between notification and mortality databases (2016–2017) identified a corrected chikungunya lethality rate ~6.8-fold higher than estimates based on the notification system alone, and chikungunya was not mentioned on most death certificates among linked deaths [43]. In a matched cohort study and self-controlled case series nested within the 100 Million Brazilian Cohort (2015–2018), Cerqueira-Silva et al. reported an elevated risk of death after chikungunya illness, particularly among older adults and individuals with comorbidities [44]. A nationwide cohort analysis in Brazil (2015–2024) further documented substantial hospitalization burden, mortality, and years of life lost associated with chikungunya, reinforcing that fatal outcomes are not limited to rare case reports and may be underestimated when surveillance relies only on laboratory-confirmed attribution [45]. Evidence from active epidemiological surveillance, including both classic autopsies and minimally invasive tissue sampling, further supports the conclusion that many chikungunya-related fatalities go undetected during clinical care [46]. This underreporting likely reflects clinical challenges in recognizing fatal cases—many driven by underdiagnosed complications such as myocarditis, encephalitis, or multi-organ failure, or indirectly by decompensation of pre-existing conditions [3,7,46,47,48,49,50]—combined with a persistent perception of chikungunya as a largely non-lethal disease, sometimes echoed in public health communications [51,52,53]. Consequently, although evidence from postmortem studies—including viral antigen immunolocalization in target organs—along with case series, case–control investigations, and immunopathological studies consistently indicates that CHIKV can contribute to death, these severe forms are not routinely recognized by healthcare professionals, leading to missed diagnoses, misclassification, and underreporting [3,7,46,47,48,49,50]. Systematic incorporation of autopsies (conventional or minimally invasive tissue sampling) and syndromic surveillance strategies, alongside excess mortality analysis as a complementary approach, could provide a more accurate and conclusive understanding of chikungunya-related mortality in future epidemics. This integrated approach would strengthen risk assessment and public health response by more accurately capturing the full impact of arboviral epidemics and outbreaks caused by newly emerging viruses.

Limitations of this study include the use of preliminary mortality data from INSEE, which may be subject to later revisions. The analysis did not account for the potential mitigating effect of the vaccination campaign conducted during the outbreak, as detailed official data on vaccine coverage were not accessible, which may have influenced mortality patterns. Additionally, other circulating pathogens or extreme natural events were not incorporated into the models and could have contributed to mortality fluctuations; however, according to French surveillance data, there was no significant transmission of other arboviruses (e.g., dengue, Zika), respiratory viruses (COVID-19, influenza, RSV), or other monitored pathogens in the region during the study period [12]. The relatively small population size of Réunion Island and the wide age group classifications may have limited the ability to detect subtle increases in mortality across specific strata, potentially leading to underdetection of age-specific excess deaths. Finally, as with all ecological studies, these analyses rely on aggregate data, which does not allow for individual-level causal inference and the assessment of pre-existing conditions’ contribution to the death outcome; therefore, the possibility of residual confounding cannot be excluded.

This recent epidemic in a French overseas department underscores the broader and evolving risk of chikungunya transmission affecting European public health, while mainland Europe is also experiencing an unprecedented pattern of early and geographically widespread autochthonous transmission. Consistent with this changing risk landscape, recent work has highlighted Italy as a European forerunner for local transmission risk assessment and preparedness planning [54]. The widespread presence of *Aedes albopictus* populations, the circulation of a highly adapted ECSA-2 viral lineage carrying the E1-A226V, E2-I211T, and E2-L210Q mutations, and the record-breaking heat experienced in several European countries in June 2025 have likely acted synergistically to create favorable conditions for early and widespread transmission [8,9,11]. Notably, autochthonous transmission was documented at latitudes as high as 48° N in Jun—an atypical occurrence for CHIKV and the earliest recorded in mainland Europe. By mid-July, 13 localities in Italy and France had reported 31 confirmed cases, already surpassing the cumulative number of affected areas documented between 2007 and 2024 [11]. Guangdong Province, China, faced its worst documented chikungunya epidemic in July 2025, with thousands of locally transmitted cases reported [10]. This unprecedented global scenario—with simultaneous outbreaks in multiple European localities at northernmost latitudes ever recorded for chikungunya and the most severe epidemic ever observed in East Asia—in the global expansion of CHIKV.

The rapid geographic spread, coupled with the historical under-recognition of the mortality burden, reinforces the urgent need to integrate excess mortality surveillance into arboviral monitoring frameworks and to consider preventive vaccination strategies in at-risk regions. These concurrent events underscore that climate anomalies, widespread vector presence, and viral adaptations can jointly amplify transmission risk far beyond historically observed patterns. Such evolving risk scenarios strongly reinforce the need to adopt a comprehensive preparedness approach that combines surveillance innovations, vaccination programs, and improved clinical care for vulnerable populations.

A recent clinical consensus statement by the European Society of Cardiology, “*Vaccination as a new form of cardiovascular prevention*” [55], illustrates the importance of explicit recognition by leading medical societies that indirect mortality—such as cardiovascular deaths triggered or exacerbated by infections—must be properly acknowledged. Such recognition is essential to ensure that medical professionals, other health workers, patients, and policymakers can deliver proportionate and timely responses.

The same logic applies to chikungunya: without formal acknowledgment of both its direct and indirect fatal burden, surveillance systems will continue to underestimate its true impact, ultimately delaying appropriate clinical and public health interventions.

## 5. Final Comments: Immediate Priorities for Action

The persistent under-recognition of chikungunya-associated mortality continues to hinder an effective epidemic response. There is an urgent need to reassess the outdated narrative of chikungunya as a non-lethal disease, prioritize research, improve diagnostics, expand postmortem investigations, and ensure the development of surveillance methods capable of capturing both direct and indirect fatal outcomes. In parallel, licensed chikungunya vaccines are now available in some settings, and vaccination strategies should be explicitly considered within preparedness frameworks—particularly for older adults and other high-risk groups in areas with established Aedes vectors and recurrent outbreaks.

The recently launched WHO Integrated Guidelines on the Clinical Management of Arboviral Diseases [56] represent an important step forward, recommending the adoption of syndromic surveillance and management approaches to improve case detection in settings where dengue, chikungunya, Zika, and yellow fever may circulate simultaneously and diagnostic capacity is limited. This approach can enhance sensitivity for early identification of severe cases and improve patient outcomes, particularly in vulnerable populations.

Aligned with these recommendations, active syndromic surveillance of severe cases and deaths is urgently needed, supported by classical autopsies and minimally invasive tissue sampling (MITS) to clarify causes of death that often go unrecognized in routine systems. Furthermore, real-time excess mortality analyses should be fully integrated into arboviral surveillance frameworks, providing actionable evidence for decision-makers and enabling timely, proportionate interventions to reduce preventable deaths during future epidemics.

## 6. Ethical Statements and Considerations

All data used in this analysis were obtained from publicly available official sources provided by French governmental institutions. We would like to express our appreciation for the transparency, timeliness, and public accessibility of these data, which reflect the French government’s commitment to open data and evidence-based public health action. This study did not involve individual-level data or human subjects and therefore did not require ethical approval.

Our research group has been systematically investigating chikungunya-associated excess mortality across different countries and regions using similar methodologies based on officially reported aggregate data. This approach is not intended to identify or expose shortcomings in local surveillance systems but rather to explore broader mortality impacts that may not be fully captured by case-based surveillance—particularly given the inherent complexity of attributing deaths that occur after variable delays following infection.

Mortality attribution related to chikungunya remains a global challenge, as delayed or indirect deaths may occur days, weeks, or even months after the initial illness, often resulting from diverse clinical complications affecting multiple organ systems. This complexity hinders individual-level attribution.

Routine surveillance systems in Réunion Island are notably comprehensive, integrating multiple data sources and ranking among the most advanced worldwide. Nonetheless, like all surveillance systems, they may face intrinsic limitations in capturing the full spectrum of chikungunya-related mortality. This study aims to contribute to the scientific understanding of this public health challenge, without criticism of local surveillance efforts or public health authorities.

We hope that these materials contribute to a transparent, reproducible, and evidence-based understanding of chikungunya-related mortality and support improved surveillance and epidemic preparedness strategies globally.

## 7. Transparency and Reproducibility

The SPSS syntax files used in the analyses, together with Python scripts (version 3.13, 64-bit, using numpy, pandas, scipy.stats, and statsmodels), as well as all source data, model outputs, and analytical spreadsheets, are publicly available in our GitHub repository [57], [https://github.com/aribasfreitas/chikungunya, accessed on 21 July 2025].

## Figures and Tables

**Table 1:** Monthly Observed and Expected Deaths, Excess Mortality, and IRR_m_ by Age Group in Réunion Island (September 2024–May 2025).

Month	Age (years)	Observed Deaths	Expected Deaths	95% CI	Excess Deaths	IRR_m_	95% CI IRR_m_ (Poisson)	95% CI IRR_m_ (Byar)	One–Tailed *p*–Value (Poisson)	Two–Tailed *p*–Value (Poisson)
**2024-09**	**<25**	10	13	[7–21]	—	0.75	[0.4–1.39]	[0.36–1.38]	0.855	0.448
**2024-10**	**<25**	15	9	[4–16]	—	1.6	[0.96–2.65]	[0.89–2.64]	0.055	0.110
**2024-11**	**<25**	12	16	[9–24]	—	0.76	[0.43–1.34]	[0.39–1.33]	0.862	0.414
**2024-12**	**<25**	10	15	[8–23]	—	0.65	[0.35–1.22]	[0.31–1.2]	0.939	0.210
**2025-01**	**<25**	11	10	[5–17]	—	1.06	[0.59–1.92]	[0.53–1.9]	0.462	0.923
**2025-02**	**<25**	10	12	[6–20]	—	0.81	[0.44–1.51]	[0.39–1.49]	0.785	0.628
**2025-03**	**<25**	12	14	[7–22]	—	0.87	[0.49–1.53]	[0.45–1.52]	0.723	0.756
**2025-04**	**<25**	7	8	[3–14]	—	0.89	[0.42–1.86]	[0.36–1.83]	0.673	0.937
**2025-05**	**<25**	11	9	[4–16]	—	1.17	[0.65–2.12]	[0.59–2.1]	0.338	0.676
**2024-09**	**25–64**	117	107	[87–128]	—	1.09	[0.91–1.31]	[0.91–1.31]	0.176	0.352
**2024-10**	**25–64**	120	111	[91–132]	—	1.08	[0.9–1.29]	[0.9–1.29]	0.2054	0.411
**2024-11**	**25–64**	114	97	[78–117]	—	1.17	[0.97–1.41]	[0.97–1.41]	0.054	0.107
**2024-12**	**25–64**	114	111	[91–133]	—	1.02	[0.85–1.23]	[0.84–1.23]	0.414	0.829
**2025-01**	**25–64**	105	114	[93–135]	—	0.92	[0.76–1.12]	[0.75–1.12]	0.809	0.436
**2025-02**	**25–64**	111	99	[80–119]	—	1.12	[0.93–1.35]	[0.92–1.35]	0.121	0.242
**2025-03**	**25–64**	100	103	[83–123]	—	0.97	[0.8–1.18]	[0.79–1.18]	0.623	0.831
**2025-04**	**25–64**	106	100	[81–120]	—	1.06	[0.87–1.28]	[0.87–1.28]	0.298	0.596
**2025-05**	**25–64**	98	108	[88–129]	—	0.91	[0.75–1.11]	[0.74–1.11]	0.840	0.370
**2024-09**	**65+**	362	388	[350–427]	—	0.93	[0.84–1.03]	[0.84–1.03]	0.911	0.196
**2024-10**	**65+**	369	402	[363–442]	—	0.92	[0.83–1.02]	[0.83–1.02]	0.953	0.104
**2024-11**	**65+**	340	351	[315–389]	—	0.97	[0.87–1.08]	[0.87–1.08]	0.7343	0.567
**2024-12**	**65+**	358	408	[369–448]	—	0.88	[0.79–0.97]	[0.79–0.97]	0.994	0.013
**2025-01**	**65+**	433	416	[377–457]	—	1.04	[0.95–1.14]	[0.94–1.14]	0.211	0.422
**2025-02**	**65+**	349	357	[321–395]	—	0.98	[0.88–1.09]	[0.88–1.09]	0.674	0.692
**2025-03**	**65+**	397	344	[308–380]	53	1.16	[1.05–1.27]	[1.04–1.27]	<0.0016	<0.01
**2025-04**	**65+**	442	330	[295–366]	112	1.34	[1.22–1.47]	[1.22–1.47]	<0.001	<0.001
**2025-05**	**65+**	403	360	[323–398]	43	1.12	[1.01–1.23]	[1.01–1.23]	0.05	<0.05
**Total excess deaths**					**208**					

IRR_m_ (ratio of observed to expected monthly death); CI (95% confidence interval); excess deaths are the observed deaths subtracting the expected deaths in the months in which lower limit CI of IRR_m_ is greater than 1 (highlighted by underlining).

**Table 2:** Monthly Observed and Expected Deaths, Excess Mortality, and Z-scores by Age Group in Réunion Island (September 2024–May 2025), Based on Poisson Regression Models.

			Serfling-Type Model					Nominal Model				
Month	Age (years)	Observed Deaths	Expected	Expected − 2SD	Expected + 2SD	Excess Deaths	Z-Score	Expected	Expected − 2SD	Expected + 2SD	Excess Deaths	Z-Score
**2024-09**	**<25**	10	10	4.3	17.1	—	0.00	9	3.6	15.8	—	0.32
**2024-10**	**<25**	15	11	5.3	18.8	—	1.15	11	5.1	18.2	—	1.15
**2024-11**	**<25**	12	10	4.3	17.1	—	0.60	10	4.4	17.0	—	0.61
**2024-12**	**<25**	10	12	6.1	20.1	—	−0.60	12	5.9	19.4	—	−0.61
**2025-01**	**<25**	11	12	6.1	20.1	—	−0.30	12	5.9	19.4	—	−0.30
**2025-02**	**<25**	10	9	4.0	15.9	—	0.00	9	3.6	15.8	—	0.32
**2025-03**	**<25**	12	12	6.1	20.1	—	0.00	12	5.9	19.4	—	0.00
**2025-04**	**<25**	7	11	5.1	18.2	—	−1.30	11	5.1	18.2	—	−1.31
**2025-05**	**<25**	11	8	3.0	15.1	—	0.95	9	3.6	15.8	—	0.63
**2024-09**	**25–64**	117	110	90.3	130.9	—	0.68	110	90.4	130.9	—	0.68
**2024-10**	**25–64**	120	111	90.4	132.0	—	0.88	112	92.2	133.0	—	0.78
**2024-11**	**25–64**	114	107	87.5	127.8	—	0.69	106	86.6	126.7	—	0.79
**2024-12**	**25–64**	114	115	94.3	136.4	—	−0.10	116	96.0	137.2	—	−0.19
**2025-01**	**25–64**	105	111	90.4	132.0	—	−0.50	109	89.4	129.8	—	−0.40
**2025-02**	**25–64**	111	97	79.1	116.3	—	1.47	97	78.2	117.1	—	1.41
**2025-03**	**25–64**	100	107	87.5	128.7	—	−0.71	108	88.5	128.8	—	−0.80
**2025-04**	**25–64**	106	101	81.9	121.4	—	0.50	100	81.0	120.3	—	0.60
**2025-05**	**25–64**	98	109	88.5	129.8	—	−1.02	110	90.4	130.9	—	−1.21
**2024-09**	**65+**	362	371	332.2	411.2	—	−0.46	374	335.3	414.1	—	−0.61
**2024-10**	**65+**	369	393	352.3	434.5	—	−1.12	391	351.7	431.7	—	−1.11
**2024-11**	**65+**	340	347	309.1	386.3	—	−0.36	348	310.2	387.2	—	−0.42
**2024-12**	**65+**	358	365	325.4	405.6	—	−0.31	365	326.6	404.8	—	−0.36
**2025-01**	**65+**	433	400	359.3	442.0	—	1.63	400	360.4	441.0	—	1.62
**2025-02**	**65+**	349	343	307.4	380.9	—	0.30	343	305.4	382.0	—	0.31
**2025-03**	**65+**	397	355	316.4	396.0	42	2.04	357	318.9	396.5	40	2.02
**2025-04**	**65+**	442	338	300.5	377.0	104	5.19	334	296.8	372.7	108	5.42
**2025-05**	**65+**	403	347	308.5	387.4	56	2.76	353	315.0	392.4	50	2.53
**Excess deaths**						**202**					**198**	

2 SD, two standard deviations from the mean. Excess deaths represent the difference between observed and expected deaths for months when observed deaths exceeded the expected mean by more than 2 SD (highlighted by underlining).

## Data Availability

The SPSS syntax files used in the analyses, together with Python scripts (version 3.13, 64-bit, using numpy, pandas, scipy.stats, and statsmodels), as well as all source data, model outputs, and analytical spreadsheets, are publicly available in our GitHub repository [57], [https://github.com/aribasfreitas/chikungunya, accessed on 21 July 2025].

## Supplementary Materials

The following supporting information can be downloaded at: https://www.mdpi.com/article/10.3390/v18020180/s1.. A detailed comparative analysis provided in the Supplementary Materials demonstrated substantial discrepancies between excess mortality and officially reported chikungunya deaths across multiple epidemics worldwide. Even in high-income territories with structured surveillance systems (e.g., Réunion Island in 2006 and 2024–2025 and French Caribbean in 2014), most deaths were only mentioned on death certificates and rarely confirmed by laboratory testing. In low- and middle-income settings, the gap between excess and reported deaths was far larger, with several epidemics showing thousands of unexplained excess deaths and no officially acknowledged fatalities. These findings underscore the limitations of traditional surveillance in quantifying chikungunya mortality and highlight the value of excess mortality analyses as a critical complementary tool for epidemic risk assessments.
