# Supplementary material for: Increased Mortality Rates During the 2025 Chikungunya Epidemic in Réunion Island
Source: Viruses. 2026 Jan 29;18(2):180. doi: 10.3390/v18020180 (PMC12944855; doi:10.3390/v18020180)
Supplement: Supplementary file 1 [file viruses-18-00180-s001.zip › viruses-3829984-supplementary.pdf]

Supplementary Material - Increased Mortality Rate during the 2025 Chikungunya Epidemic in Réunion Island

André Ricardo Ribas Freitas

Faculdade São Leopoldo Mandic, Campinas 13045, SP , Brazil:
andre.freitas@slmandic.edu.br

Luana Hughes Freitas

Faculdade de Engenharia de Computação. Insper, Rua Quatá, 300, Vila Olímpia. CEP: 04546-042 São Paulo – SP, Brazil:
luanahf1234@gmail.com

Antônio Silva Lima Neto

Centro de Ciências da Saúde. Universidade de Fortaleza , Fortaleza 60811, CE, Brazil:
tanta26@yahoo.com

Luciano Pamplona Goes Cavalcanti

Faculdade de Medicina, Centro Universitário Christus, Fortaleza 60160, CE, Brazil:
pamplona.luciano@gmail.com

Pedro María Alarcón-Elbal

Faculty of Veterinary Medicine, Universidad Cardenal Herrera-CEU, CEU Universities, Calle Tirant lo Blanc 7, Alfara del Patriarca, 45115 Valencia, Spain:
pedro.alarconelbal@uchceu.es

1. Excess Mortality Versus Officially Reported Deaths Attributed to Chikungunya: Mini Review of Multi-Country Analysis

This supplementary section presents a comparative analysis between officially reported chikungunya-related deaths and estimates of excess mortality during major epidemics in multiple countries and territories. Excess mortality figures were retrieved from peer-reviewed publications and official surveillance reports and represent the number of deaths exceeding expected baseline levels during defined epidemic periods. Reported deaths were systematically extracted from official documents, including government epidemiological bulletins, ministry of health reports, and international surveillance databases. These figures reflect deaths officially attributed to chikungunya, whether through laboratory confirmation, clinical-epidemiological criteria, or mention of chikungunya on the death certificate.

The ratio of excess to reported deaths was calculated to estimate the magnitude of underreporting. Where available, the case classification criteria and circulating viral lineage were documented to provide contextual insights. Table 3 compiles data from various outbreaks, highlighting major discrepancies between excess mortality and officially reported fatalities.

Table S1 compiles data from multiple chikungunya epidemics worldwide, illustrating striking differences between excess mortality estimates and the number of deaths officially reported. The patterns observed indicate that underrecognition of chikungunya mortality is a consistent phenomenon, albeit with substantial heterogeneity across regions and epidemic years. Across the epidemics analyzed, underascertainment of chikungunya deaths was widespread, with significant variation by geographic and socioeconomic setting.

Even in high-income territories with relatively well-structured surveillance systems, such as Réunion Island and the French Caribbean, official statistics captured only a fraction of the true fatal burden, and laboratory-confirmed deaths were particularly low. During the 2006 Réunion epidemic, for example, 260 excess deaths were estimated, yet only 255 deaths were mentioned on death certificates and just 67 were laboratory-confirmed among hospitalized patients—representing roughly one quarter of the excess mortality estimate.

Similarly, during the 2014 epidemic in Martinique and Guadeloupe, 639 excess deaths were estimated, while only 160 deaths appeared in official reports, and fewer than half of these (74) were supported by laboratory confirmation. In both events—the 2006 Réunion outbreak and the 2014 French Caribbean epidemic—the majority of recognized deaths were not based on laboratory evidence but rather on mention of chikungunya on death certificates.

This underascertainment persisted in the most recent large epidemic in Réunion Island (2024–2025, present study). Despite a well-established health information system, an estimated 208 excess deaths occurred, yet only 17 were officially reported as directly attributable to chikungunya, and just 27 were recognized when indirect causes were included. This represents an undercount of up to twelve-fold relative to excess mortality estimates. These findings highlight that, despite advances in surveillance and diagnostic

capacity over the past two decades, the proportion of laboratory-confirmed fatal cases has remained remarkably low, revealing enduring gaps in the ability of health systems to detect and accurately attribute chikungunya mortality.

In many low- and middle-income settings, underreporting was even more severe or absolute. Ahmedabad (India, 2006) saw over 2,900 excess deaths with zero officially recognized fatalities. Similar patterns were observed in Port Blair and Mauritius, where 86 and 743 excess deaths respectively occurred without a single confirmed death. The Dominican Republic had one of the starkest discrepancies ever documented: 4,925 excess deaths compared to just six reported fatalities—a ratio exceeding 800. Even Puerto Rico, despite a relatively robust health information system, recorded 42 times fewer deaths than the estimated excess (1,310 excess vs. 31 reported). In Jamaica, nearly 2,500 excess deaths were observed during the 2014 epidemic, yet no deaths were officially acknowledged.

Brazil presented a heterogeneous scenario. In several states during 2015–2016, reported deaths captured only a small proportion of the estimated excess mortality. For example, in Pernambuco, 4,505 excess deaths were estimated during the 2016 epidemic, yet only 94 were reported as chikungunya-related. In Rio Grande do Norte, 1,478 excess deaths occurred with only 64 deaths reported. Similar patterns were observed in Bahia and Minas Gerais, where laboratory and clinical-epidemiological criteria identified few cases compared to large estimated excesses, leading to ratios as high as 155 in some settings.

Overall, these findings demonstrate that the gap between excess and reported mortality is not confined to resource-limited countries. Even in territories with well-established health systems, a substantial proportion of chikungunya-associated deaths—especially those with laboratory confirmation—remains unrecognized in official statistics. This undercount reflects persistent limitations in traditional surveillance methods, which struggle to capture the true mortality burden of chikungunya, particularly during large-scale epidemics that overwhelm health services.

Excess mortality analysis emerges as an essential complementary tool for risk assessment and public health response, capable of uncovering the hidden fatal toll of chikungunya that conventional surveillance fails to detect, regardless of a country's income level or healthcare infrastructure.

Table S1. Comparison of excess deaths and reported chikungunya deaths during epidemics in different countries

Country	Location	Year	Poputation of studied area (X1,000)	Excess deaths (1)	Excess mortality rate (/100,000)	Reported deaths (2)	Classification criteria	Excess/ Reported Ratio (1/2)	Probable/ confirmed lineage	Source_(excess_deaths)
**French**	**Reunion**	**2006**	770	260	33.8	255	Mention of chikungunya on the death certificate[1]	1.02	ECSA (IOL)	Bull épid hebdom thém, 2008[1]
						67	Lab-confirmed hospitalized cases[1]	3.9		
	**Martinica & Guadaloupe**	**2014**	783	639	81.6	160	Not documented (N.D.) [2]	4.0	Asian	Epid & Infect, 2018[3]
						74	Lab-confirmed hospitalized cases[2]	8.6		
	**Reunion**	**2024**	896	208	23.2	17	Lab-confirmed (Direct cause)[4]	12.2	ECSA-2	Present study,2025
						27	Lab-confirmed (direct + Indirect cause)[4]	7.7		
**India**	**Calcutta**	**1963**	N.D.	158	N.D.	10	diagnosed clinically hospitalized cases[5]	15.9	N.D.	Report of WHO/ SEA/WPR Seminar on Mosquito-borne Haemorrhagic Fevers[6]
						2	viral isolation hospitalized cases[5]	78	N.D.	
	**Ahmedabab**	**2006**	3,800	2944	77.5	0	Not reported	...	ECSA	EID, 2008[7]
	**Port Blair**	**2006**	136	86	63.2	0	Not reported	...	ECSA	Epid & Infect, 2011[8]
**Mauritius**		**2006**	1,250	743	59.4	0	Not reported	...	ECSA (IOL)	EID, 2008[9]
**Puerto Rico**		**2014**	3,688	1310	35.5	31	Lab-confirmed[10]	42.3	Asian	EID, 2018[11]
**Dominican Republic**		**2014**	10,400	4925	47.4	6	N.D.[12]	820.8	Asian	TRSTMH 2018[13]
**Jamaica**		**2014**	2,720	2499	91.9	0	Not reported	...	Asian	Pat & Glob Health, 2019[14]
**Brazil**	**Bahia**	**2015**	2273	270	11.9	12	Lab-confirmed[15]	22.5		PLoS Currents, 2017[16]
	**Bahia**	**2016**	6,853	1,247	18.2	8	Lab-confirmed[15]	155.9		PLoS Currents, 2017[16]
	**Pernambuco**	**2016**	9,410	4,505	47.9	94	Lab-confirmed[15]	47.9	ECSA	PLoS Currents, 2017[16]
	**Rio Grande do Norte**	**2016**	3,474	1,478	42.5	64	Lab-confirmed[15]	23.1	ECSA	PLoS Currents, 2017[16]
	**Minas Gerais**	**2023**	2,535	819	32.3	15	Laboratory (10), clinical-epidemiological (5)[15]	54.6	ECSA	Frontiers Trop Med, 2024[17]
**Total**			**48,988**	**21,933**	**44.77**	**829**		**26.5**		

Reported deaths" refer to those officially registered as caused by chikungunya, either through laboratory confirmation, clinical-epidemiological criteria, or mention on death certificates.

2. Ethical Statements and Considerations

All data used in this analysis were obtained from publicly available official sources provided by French governmental institutions. We would like to express our appreciation for the **transparency, timeliness, and public accessibility** of these data, which reflect the French government's commitment to open data and evidence-based public health action. The study did not involve individual-level data or human subjects, and therefore did not require ethical approval.

Our research group has been systematically investigating chikungunya-associated excess mortality across different countries and regions, using similar methodologies based on officially reported aggregate data. This approach is not intended to identify or expose shortcomings in local surveillance systems, but rather to explore broader mortality impacts that may not be fully captured by case-based surveillance—particularly given the inherent complexity of attributing deaths that occur after variable delays following infection.

Mortality attribution related to chikungunya remains a global challenge, as delayed or indirect deaths may occur days, weeks, or even months after the initial illness, often resulting from diverse clinical complications affecting multiple organ systems. This complexity hinders individual-level attribution.

Routine surveillance systems in Réunion Island are notably comprehensive, integrating multiple data sources and ranking among the most advanced worldwide. Nonetheless, like all surveillance systems, they may face intrinsic limitations in capturing the full spectrum of chikungunya-related mortality. This study aims to contribute to the scientific understanding of this public health challenge, without criticism of local surveillance efforts or public health authorities.

Bibliography

1 Santé Publique France. Bulletin Epidémiologique Hebdomadaire, 21 octobre 2008, n°38-39-40 Qu'avons-nous appris de l'épidémie de chikungunya dans l'Océan Indien en 2005-2006 ? 2008. https://www.santepubliquefrance.fr/import/bulletin-epidemiologique-hebdomadaire-21-octobre-2008-n-38-39-40-qu-avons-nous-appris-de-l-epidemie-de-chikungunya-dans-l-ocean-indien-en-2005-20 (accessed July 24, 2025).

2 Santé Publique France. Situation épidémiologique du chikungunya dans les Antilles. Point au 18 mai 2015. 2015. https://www.santepubliquefrance.fr/guyane/situation-epidemiologique-du-chikungunya-dans-les-antilles.-point-au-18-mai-2015 (accessed July 24, 2025).

3 Ribas Freitas, A. R., Alarcón-Elbal PM, Donalisio MR. Excess mortality in Guadeloupe and Martinique, islands of the French West Indies, during the chikungunya epidemic of 2014. Epidemiology and Infection. 2018; **146**: 2059–65.

4 Santé Publique France. Surveillance sanitaire à La Réunion. Bulletin du 26 juin 2025. 2025. https://www.santepubliquefrance.fr/regions/ocean-indien/documents/bulletin-regional/2025/surveillance-sanitaire-a-la-reunion.-bulletin-du-26-juin-2025 (accessed July 23, 2025).

5 Sarkar JK, Chatterjee SN, Chakravarty SK. Three-year study of mosquito-borne haemorrhagic fever in Calcutta*. *Transactions of The Royal Society of Tropical Medicine and Hygiene* 1967; **61**: 725–35.

6 Pacific WHORO for the W. Report of the WHO Seminar on Mosquito-borne Haemorrhagic Fevers in South-East Asia and Western Pacific Regions, Bangkok, 19-26 October 1964. 1964. https://iris.who.int/handle/10665/207795 (accessed Aug 3, 2025).

7 Mavalankar D, Shastri P, Bandyopadhyay T, Parmar J, Ramani KV. Increased Mortality Rate Associated with Chikungunya Epidemic, Ahmedabad, India. *Emerging Infectious Diseases* 2008; **14**: 412–5.

8 Manimunda SP, Mavalankar D, Bandyopadhyay T, Sugunan AP. Chikungunya epidemic-related mortality. *Epidemiology & Infection* 2011; **139**: 1410–2.

9 Beesoon S, Funkhouser E, Kotea N, Spielman A, Robich RM. Chikungunya Fever, Mauritius, 2006. *Emerging Infectious Disease* 2008; **14**: 337–8.

10 Sharp TM, Ryff KR, Alvarado L, *et al.* Surveillance for Chikungunya and Dengue During the First Year of Chikungunya Virus Circulation in Puerto Rico. *Journal of Infectious Diseases* 2016; **214**: S475–81.

11 Freitas ARR, Donalisio MR, Alarcón-Elbal PM. Excess Mortality and Causes Associated with Chikungunya, Puerto Rico, 2014–2015. *Emerging Infectious Diseases* 2018; **24**: 2352–5.

12 Pan American Health Organization. PAHO WHO | Chikungunya | Statistic Data. 2016. http://www.paho.org/hq/index.php?option=com_topics&view=readall&cid=5927&Itemid=40931&lang=en (accessed Dec 13, 2016).

13 Freitas ARR, Alarcón-Elbal PM, Paulino-Ramírez R, Donalisio MR. Excess mortality profile during the Asian genotype chikungunya epidemic in the Dominican Republic, 2014. *Transactions of The Royal Society of Tropical Medicine and Hygiene* 2018; **112**: 443–9.

14 Freitas ARR, Gérardin P, Kassar L, Donalisio MR. Excess deaths associated with the 2014 chikungunya epidemic in Jamaica. *Pathogens and Global Health* 2019; : 1–5.

15 Série Histórica - Casos óbitos chikungunya (2015-2023) — Ministério da Saúde. https://www.gov.br/saude/pt-br/assuntos/saude-de-a-a-z/c/chikungunya/situacao-epidemiologica/serie-historica-casos-obitos-chikungunya-2015-2023/view (accessed July 24, 2025).

16 Freitas ARR, Cavalcanti L, Zuben APV, Donalisio MR. Excess Mortality Related To Chikungunya Epidemics In The Context Of Co-circulation Of Other Arboviruses In Brazil. *PLOS Currents Outbreaks* 2017; : 140491.

17 Ribas Freitas AR, Lima Neto AS, Rodrigues R, Alves de Oliveira E, Andrade JS, Cavalcanti LPG. Excess mortality associated with chikungunya epidemic in Southeast Brazil, 2023. *Front Trop Dis* 2024; **5**: 1466207.
